# P finder: genomic and metagenomic annotation of RNase P RNA gene (*rnpB*)

**DOI:** 10.1186/s12864-020-6615-z

**Published:** 2020-04-29

**Authors:** J. Christopher Ellis

**Affiliations:** Computational Biology and Bioinformatics Group, Biosciences Division, Oak Ridge National Laboratory, Oak Ridge, TN 37831 USA

**Keywords:** RNase P RNA, Ribonuclease P, rnpB, Metagenomic, Genomic, Gene annotation, Genome annotation

## Abstract

**Background:**

The *rnpB* gene encodes for an essential catalytic RNA (RNase P). Like other essential RNAs, RNase P’s sequence is highly variable. However, unlike other essential RNAs (i.e. tRNA, 16 S, 6 S,...) its structure is also variable with at least 5 distinct structure types observed in prokaryotes. This structural variability makes it labor intensive and challenging to create and maintain covariance models for the detection of RNase P RNA in genomic and metagenomic sequences. The lack of a facile and rapid annotation algorithm has led to the *rnpB* gene being the most grossly under annotated essential gene in completed prokaryotic genomes with only a 24% annotation rate. Here we describe the coupling of the largest RNase P RNA database with the local alignment scoring algorithm to create the most sensitive and rapid prokaryote *rnpB* gene identification and annotation algorithm to date.

**Results:**

Of the 2772 completed microbial genomes downloaded from GenBank only 665 genomes had an annotated *rnpB* gene. We applied P Finder to these genomes and were able to identify 2733 or nearly 99% of the 2772 microbial genomes examined. From these results four new *rnpB* genes that encode the minimal T-type P RNase P RNAs were identified computationally for the first time. In addition, only the second C-type RNase P RNA was identified in *Sphaerobacter thermophilus*. Of special note, no RNase P RNAs were detected in several obligate endosymbionts of sap sucking insects suggesting a novel evolutionary adaptation.

**Conclusions:**

The coupling of the largest RNase P RNA database and associated structure class identification with the P Finder algorithm is both sensitive and rapid, yielding high quality results to aid researchers annotating either genomic or metagenomic data. It is the only algorithm to date that can identify challenging RNAse P classes such as C-type and the minimal T-type RNase P RNAs. P Finder is written in C# and has a user-friendly GUI that can run on multiple 64-bit windows platforms (Windows Vista/7/8/10). P Finder is free available for download at https://github.com/JChristopherEllis/P-Finder as well as a small sample RNase P RNA file for testing.

## Background

Ribonuclease P (RNase P) is a ribonuclease and is comprised of an RNA (RNase P RNA) and one or more protein subunits, forming the complete holoenzyme. The RNA, not the protein, is the catalytic subunit and has been studied extensively for its role in the maturation of transfer RNA (tRNA). The RNase P holoenzyme complex hydrolyzes the phosphodiester bond in the pre-tRNA generating a 5΄-mature tRNA and smaller 5΄ tRNA leader fragment. Due to RNase P RNAs role in the maturation of tRNA it is considered an essential gene for life with only a single exception in prokaryotes described previously [1]. To date, it has been found in every Domain of life (bacteria, archaea, and eukaryote) including plastids and mitochondria and is widely believed to be a relic from the RNA world.

RNase P has multiple substrates. Though it is well studied for its role in the maturation of tRNA it can recognize and catalyze a wide variety of RNA molecules in the cell. For example, it can recognize and cleave 2 S, 4.5 S, and tmRNAs, [2–7]. In addition, it has also been shown to cleave the 5΄UTR leader sequence of some messenger RNAs (mRNAs) that contain riboswitches. Altman et al. demonstrated that RNase P can cleave the coenzyme B_12_ riboswitch in *E. coli* and *B. subtilis* [6]. Interestingly, the cleavage of the riboswitch does not appear to occur in a known secondary structure commonly associated with RNase P substrates. The authors argue, convincingly, that RNase P likely recognizes transient structural motifs of the riboswitch that enable it to process the 5΄ leader sequence of the mRNA as substrate.

Like most non-coding RNAs (ncRNAs) RNase P RNAs have high sequence variability and it is this variability that poses a challenge to properly annotating *rnpB* genes in genomic and metagenomic sequence data. In other ncRNAs, such as 16S and tRNA, sequence variability possess less of an identification and annotation hurdle because they have conserved secondary structures. This conserved structure allows researchers to generate a descriptor model coupled with a covariance model for structure aided identification and annotation. Unfortunately, this is not the case with RNase P RNA.

Currently, there are at least five widely accepted RNase P RNA structures (classes) found in prokaryotes: “A” type (ancestral), “B” type (named for *Bacillus*, in which it was first described), “C” type (*Choroflexi*), “M” type (*Mehtanococci*), and “T” type (*Thermoproteaceae*). A-type RNase P RNAs are distinguished from B-type RNase P RNAs by the presence of the hairpins P6, P16, and P17 and the absences of P *P5.1, P10.1, P15.1 and P15.2* [8]*.* The C-type RNase P RNAs appear to be a structural intermediate of A and B-type RNase P RNAs [8].The C-type structural class has P6, P16, and P17 like A-type RNase P RNAs [8]. In addition, they also have structural elements found in B-types RNase P RNAs P10.1 and P15.2. M-type RNase P RNase are found exclusively in archaea and specifically, in the phyla *Methanococci and Archaeoglobi*. M-type RNase P RNAs are similar to A-type structurally except they lack P8 and L15 [9]. The minimal T-type RNase P RNA is also found only in the archaeal Order *Thermoproteaceae.* In the T-type RNAse P RNAs nearly all of the S-domain is absent *(P11, J11/12 and J12/11 including CR II and CR III, and P12)* but remains catalytically active [10].

With new structures and structural variants being described a structure aided identification and annotation approach is challenging because it requires continuous updates of both the descriptor model and covariance model. The updates to these models are time consuming, often requiring structure-based alignments by hand.

The lack of properly annotated *rnpB* genes in completed genomes is most likely from a lack of actively supported annotation software. Because of the challenges associated with properly identifying RNAse P RNA in large sequence data sets only two algorithms have been published to date: a pattern matching algorithm by Altman et al. and a covariance model algorithm called BCheck [11, 12]. Both algorithms advanced the field in the identification and annotation of RNase P RNA but neither are being actively maintained. Here the next generation of *rnpB* annotation software (P Finder) is described and compared to BCheck to demonstrate its accuracy and efficiency in detecting RNase P RNA in large sequence datasets.

## Implementation

P Finder is written in C# and has been tested in multiple 64-bit Windows environments (Windows 7/8/Vista). It has a user-friendly graphical user interface (GUI) that allows for intuitive RNase P RNA identification and/or annotation. The user can select from four search criteria including the use multiple threads. In addition, the user can determine what display data they wish to incorporate in the output such as: bitscore, E-value, percent ID, start location, end location, the strand in which the sequence is located, and of course the sequence itself. P Finder is the only available algorithm that is capable of not only identifying the RNase P RNA for annotation but also differentiating among the five different RNase P RNA structural types (A, B, C, M, and T) with the option to select this data for display in the final output.

P Finder is composed of the largest prokaryote RNase P RNA database yet assembled with more than 8300 bacterial and archaeal sequences incorporated from the RNase P Database, Rfam, and personal communications. In addition, there were several sequences identified by BCheck but not found by P Finder that have been added to the database to further enhance P Finder’s search capabilities [13, 14]. The database was also curated with the appropriate structural type for each sequence, allowing proper identification and dissemination among and between other RNase P RNA structural types.

The GUI allows the user to upload a Fasta file that contains one or more sequences to be analyzed. The size file and number of the sequences P Finder can analyze is only limited to the processing capabilities of the computer being used for *rnpB* annotation.

## Results

### To P or not to P: identifying RNase P RNA (*rnpB*) in genomic sequences

**Fig. 1:**
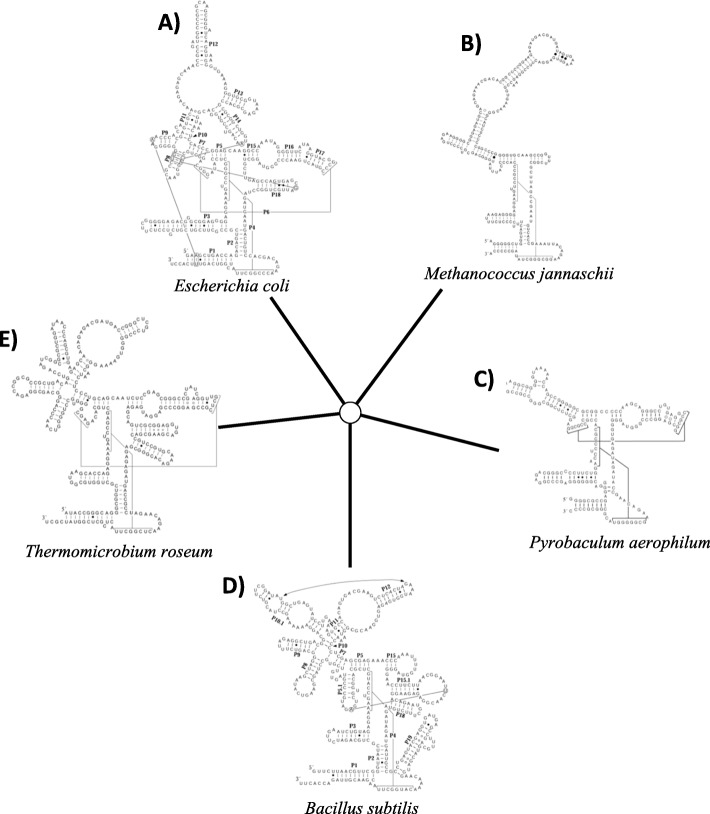
Structural variability of RNase P RNA (RNase P RNA structures were adapted with permission from the RNAse P Database [14]). RNase P RNA varies in both structure and length. **a**) *Escherichia coli* A-type **b**) *Methanococcus jannaschii* M-type **c**) *Pyrobaculum aerophilum* T-type **d**) *Bacillus subtilis* B-type **e**) *Thermomicrobium roseum* C-type RNase P RNA

RNAse P RNA (*rnpB*) is poorly annotated in completed genomic sequences available for download at GenBank. Unlike other ncRNAs RNase P RNAs the sequence length (250-550 nt) and structure (Fig. 1) are highly variable making it one of the more challenging ncRNAs to identify computationally. Of the 2772 genomes downloaded from GenBank only 665 or 24% had a *rnpB* gene annotated. Using P Finder we scanned all 2772 genomes and identified an *rnpB* gene in 2733 or nearly 99%. Comparatively, BCheck was able to identify the *rnpB* gene in 2589 or 93% of all genomes examined.

**Fig. 2:**
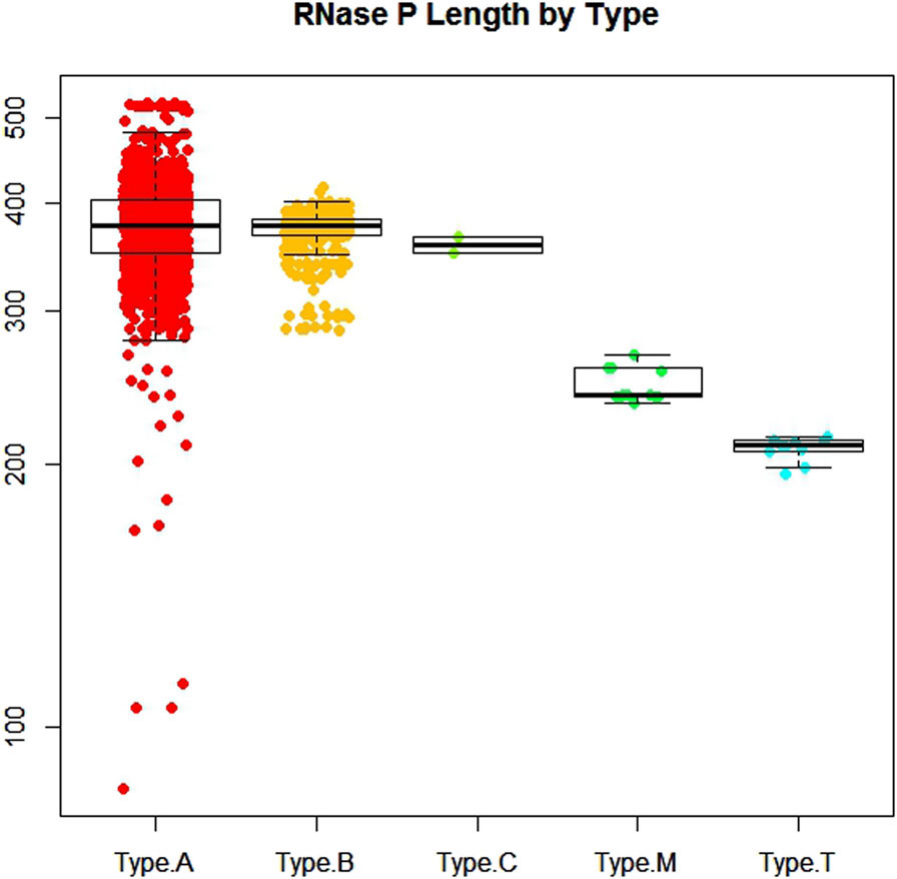
Distribution of RNase P RNA structural classes by type and length. RNase P RNA has a broad diversity of sequence length and structure. A-type RNase P RNAs are the most common structural class. The minimal T-type, C-type, and M-type are uncommon with only 28 organisms identified to date with one of these structural classes

Using these results, we examined the distribution of the different structural types in the completed genomic sequences available for download at GenBank. A-type RNase P RNAs are the most abundant with 2203 sequences or 80% of all completed genomes. They had an average sequence length of 374 nucleotides (nt) and have the greatest sequence length variability (Fig. 2). B-type was the second most common structural type observed with 504 *rnpB* genes identified. The remaining three types (C, M, and T-type RNase P RNAs) are not commonly found. To date M-type and P-type have only be observed in archaea sequences. In all, only 26 genomes encoded either a C, M, or T-type RNase P RNA. P Finder was able to identify the second C-type RNase P RNA in *Sphaerobacter thermophilus.* In addition, the minimal T-type RNase P RNAs were detected with 16 T-type RNase P RNAs identified, 10 of which were previously undescribed (Table 1).

**Table 1:** Examples of *rnpB* predictions by P Finder compared with BCheck

Structural Type	Accesion	P Finder		BCheck		Strand
		Start	End	Start	End	
**A-Type (Bacteria and Archaea)**						
trachomatis	NC_022118.1	457146	457551	457145	457548	plus
Escherichia coli LF82	NC_011993.1	3317722	3318098	3317726	3318098	minus
Klebsiella pneumoniae MGH 78578	NC_009648.1	3896359	3896741	3896363	3896741	minus
Halobacteroides halobius DSM 5150	NC_019978.1	1887825	1888178	1887824	1888177	plus
Cyanobacterium aponinum PCC 10605	NC_019776.1	1149026	1149401	1149025	1149400	plus
Candidatus Korarchaeum cryptofilum	NC_010482.1	603218	603511	603217	603510	plus
Methanobacterium sp. SWAN-1	NC_015574.1	2345793	2346086	2345792	2346085	plus
Picrophilus torridus DSM 9790	NC_005877.1	818122	818422	818121	818421	plus
Methanobacterium sp. AL-21	NC_015216.1	221739	222031	221738	222030	plus
**B-Type**						
Mycoplasma conjunctivae HRC/581	NC_012806.1	816571	816858	816570	816857	plus
CandidatusPhytoplasmaasteris	NC_007716.1	652124	652495	652117	652499	minus
Bacillus subtilis QB928	NC_018520.1	2310826	2311206	2310818	2311211	minus
Streptococcus pyogenes MGAS6180	NC_007296.1	1389794	1390162	1389786	1390167	minus
Staphylococcus aureus T0131	NC_017347.1	1533810	1534189	1533802	1534194	minus
**C-Type**						
Sphaerobacter thermophilus DSM 20745	NC_013523.1	1710922	1711286	-	-	plus
Thermomicrobium roseum DSM 5159	NC_011959.1	714587	714936	-	-	minus
**M-Type**						
Methanothermococcus okinawensis IH1	NC_015636.1	713221	713458	713220	713457	plus
Methanocaldococcus vulcanius M7	NC_013407.1	1569639	1569896	1569638	1569895	plus
Archaeoglobus fulgidus DSM 4304	NC_000917.1	86045	86279	86044	86278	plus
Methanotorris igneus Kol 5	NC_015562.1	1095229	1095484	1095228	1095483	plus
Methanocaldococcus jannaschii	NC_000909.1	643505	643762	643504	643761	plus
Methanococcus maripaludis C7	NC_009637.1	992687	992925	992686	992924	plus
Methanococcus vannielii SB	NC_009634.1	1218466	1218705	1218465	1218704	plus
Methanocaldococcus fervens AG86	NC_013156.1	1009728	1009985	1009727	1009984	plus
**T-Type**						
Caldivirga maquilingensis IC-167	NC_009954.1	1690026	1690220	-	-	plus
Pyrobaculum aerophilum str. IM2	NC_003364.1	542975	543185	-	-	plus
Pyrobaculum arsenaticum DSM 13514	NC_009376.1	124150	124362	-	-	plus
Pyrobaculum calidifontis JCM 11548	NC_009073.1	104104	104313	-	-	minus
Pyrobaculum islandicum DSM 4184	NC_008701.1	1063572	1063783	-	-	minus
Pyrobaculum neutrophilum V24Sta	NC_010525.1	114806	115020	-	-	minus
Pyrobaculum oguniense TE7	NC_016885.1	2039219	2039431	-	-	minus
Thermoproteus tenax Kra 1	NC_016070.1	1226650	1226847	-	-	minus
Thermoproteus uzoniensis 768-20	NC_015315.1	1810769	1810975	-	-	minus

**Fig. 3:**
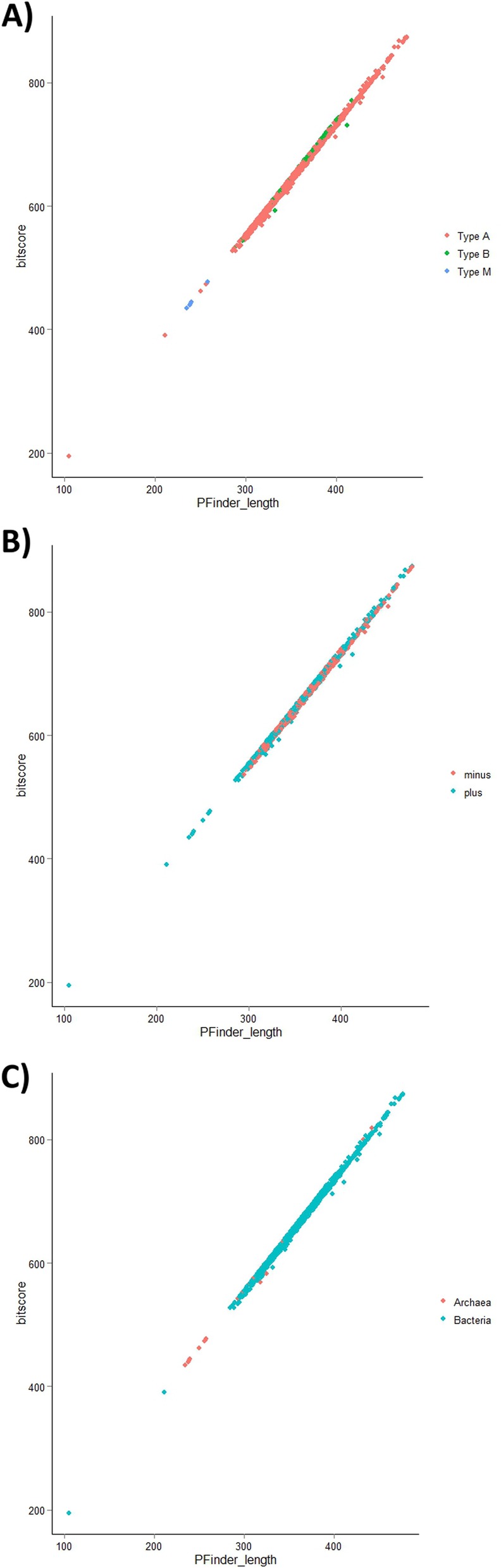
The distribution of different structural classes of RNase P RNA in genomic sequences. **a**) A plot of the different structural classes by length and quality (bitscore) identified by P Finder **b**) A plot of the distribution by strand location of *rnpB* **c**) A plot of the RNase P RNA length found in archaea and bacteria

Given RNase P RNAs have different structural variants we examined the distribution of the structural types of RNase P RNAs to determine if any relationship existed between the length of the RNAse P and type, taxonomic Kingdom, or coding strand exists. Not surprisingly, M-type and T-type RNase P RNAs are encoded only by archaea and are typically the smallest RNase P RNAs (Fig. 3). There did not appear to be any correlation between the taxonomic kingdom or the strand in which the *rnpB* gene is encoded and the length of the RNase P RNA (Fig. 3).

### Rapid and accurate RNase P RNA gene identification and annotation

**Fig. 4:**
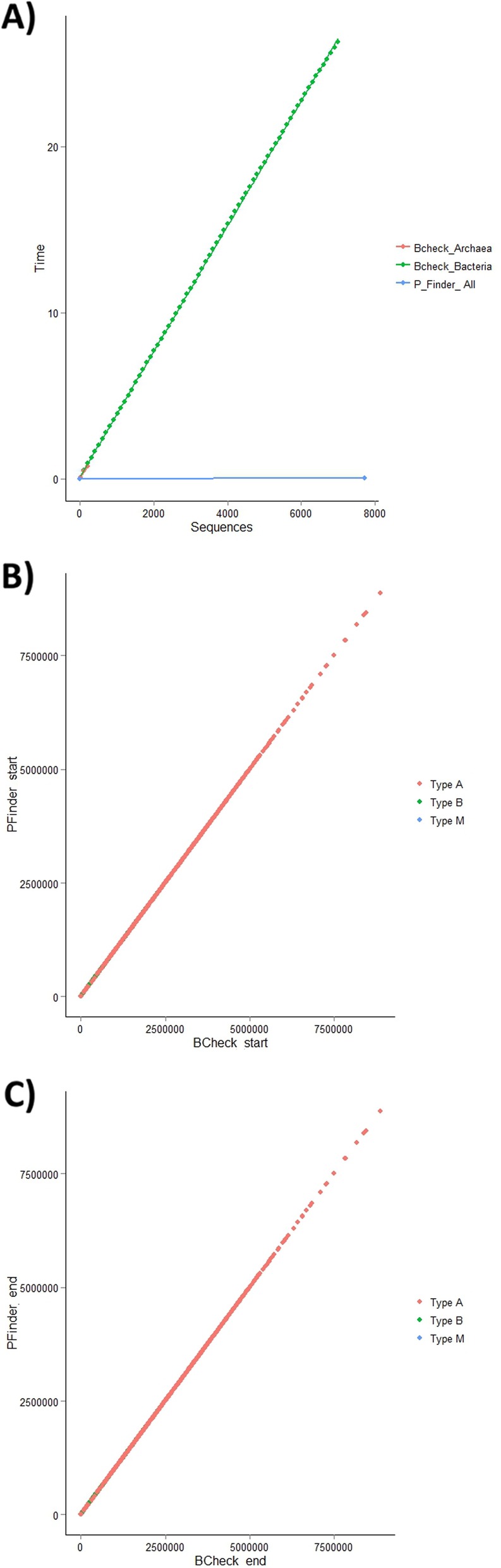
Speed comparison of P Finder with existing *rnpB* gene annotation software (BCheck). P **a**) Finder is 100X faster than currently available *rnpB* gene identification software. In addition, P Finder can differentiate between archaea RNaseP RNA and bacterial RNase P RNA eliminating the need for multiple analysis steps. P Finder demonstrates good agreement with BCheck in the prediction of start and stop locations of the rnpB gene in the genome **b**) Comparison of P Finder and BCheck’s predicted start location of the *rnpB* gene **c**) Comparison of P Finder and BCheck’s predicted end location of the *rnpB* gene

P Finder is the fastest *rnpB* gene identification and annotation software developed to date. To determine if P Finder is faster than other existing software, we downloaded BCheck. BCheck is no longer actively supported and did not work after download. We corrected some minor programming problems and ran BCheck and P Finder on a 64-bit Intel(R) Core(TM) i7-4700MQ CPU @ 2.40GHz with 16Gb of RAM. We downloaded 7531 known RNase P sequences from RFam. BCheck is not able to differentiate between archaeal and bacterial RNase P RNAs so we ran the algorithm twice, with two different settings, one for bacteria and one for arachaea. BCheck was able to complete the bacterial arm of the analysis in 26 h 16 min and 19 s and the archaea analysis in 45 min and 52 s. P Finder is able to differentiate between archaea and bacterial RNase P RNAs allowing all 7531 sequences to be run at once. P Finder completed the analysis of all the sequences in 15 min and 3 s, more than 100X faster (Fig. 4).

Though speed is important it is far more important to have a high degree of precision and accuracy. To examine P Finder’s accuracy, we compared it to BCheck by having both algorithms identify the *rnpB* gene in the 2772 completed genomes downloaded from GenBank. The predicted start and end location on the genome for both P Finder and BCheck were captured and then plotted (Fig. 4). The plots reveal good agreement between the two algorithms for both the predicted start location and the predicted ending location of the *rnpB* gene in genomic sequence. Not surprisingly, the average size of the predicted *rnpB* genes is nearly identical. The average predicted *rnpB* gene for P Finder is 372.3 nt compared to BCheck’s 373 nt. To further compare the results, we ran BLAST2 between the BCheck’s and P Finder genomic results. The average percent identity was 100% and the average bit-score was 675.5 demonstrating good agreement between the two approaches.

Though there is good agreement between P Finder and BCheck in predicted *rnpB* genes of A, B, and M-type. The most variation of predicted *rnpB* genes occurs among the B-type RNase P RNAs. BCheck appears to identify the start location a few nucleotides before P Finder. Also, BCheck predicts the end location a few nucleotides after P Finder. Though the size difference is trivial the addition of these nucleotides at the start and at the end of the BCheck’s predicted *rnpB* is likely due to an elongation of the P1 hairpin. (Fig. 4c) It is unlikely, that this is biologically relevant but further wet lab work may be needed to be able to accurately predict the correct length of the P1 hairpin in some B-type RNase P RNAs (Table 1).

### The enigma of P

RNase P RNA performs an important cellular function, the maturation of tRNA. In the absence of RNase P RNA cells are no longer capable of producing mature tRNAs and by extension unable to translate mRNAs into proteins. For this reason, RNase P RNA is considered an essential gene. One prokaryote that has been demonstrated to survive without RNase P RNA is the archaea *Nanaoarchaeum equitans. N. equitans* is able to survive without RNase P RNA by transcribing its tRNAs as mature tRNAs not needing further processing by RNase P [1]. Another prokaryote *Aquifex aeolicus*, a deep branching hyperthermophilic bacterium added to the enigma of RNase P. Researchers were unable to detect a RNase P RNA or an *rnpA* protein component until Nickel et al. described the first bacterial protein only RNase P. The newly described protein consists of a novel 23-kDa polypeptide comprised of a metallonuclease domain only and is found in some bacteria and many archaea [15].

Neither P finder nor BCheck were able to identify an *rnpB* gene in several bacterial obligate endosymbionts (Table 2). These organisms have extremely small genomes typically less than 400,000 base pairs. In fact, due to their limited genome size some researchers question if they are more organelle than bacteria. All of these microorganisms live in sap sucking insects and provide the insect with needed nutrients for survival. However, with this condensed genome there have been many genes deleted and one appears to be the *rnpB* gene.

**Table 2:** Life without P? A list of microorganisms in which no RNase P RNA can be identified.

No Rnase P RNA?	Genome size (nt)	endosymbiont of…
Candidatus Carsonella ruddii uid58773	159, 662	*Pachpsylla venusta*
Candidatus Hodgkinia cicadicola Dsem uid59311	144,000	Cicadas
Candidatus Nasuia deltocephalinicola NAS ALF uid214084	112,000	mealy bugs
Candidatus Portiera aleyrodidarum BT B uid173859	357,000	*Bemisia tabaci*
Candidatus Tremblaya phenacola PAVE uid209173	170,00	mealy bugs
Candidatus Tremblaya princeps PCIT uid68741	138,000	mealy bugs
Candidatus Uzinura diaspidicola ASNER uid186740	263, 000	armoured scale insects
Candidatus Zinderia insecticola CARI uid52459	208,000	*Clastoptera arizonana*

In bacteria, the RNase P holoenzyme is composed of the RNase P RNA and a single protein subunit (*rnpA*). We scanned the genomes of these obligate endosymbionts for the *rnpA* gene. However, we did not find an annotated *rnpA* gene in any of these eight genomes. The *rnpA* gene is part of a conserved genomic arrangement in many bacteria first analyzed by Hartmann & Hartmann [16]. It is located immediately downstream of the *rmpH* gene and immediately upstream of the *yidD* gene [17–19]. We then searched these eight genomes for this conserved genomic arrange but were unable to find any of the three genes (*rmpH*, *rnpA*, or *yidD*) in this conserved genomic arrangement. The absence of these three genes in the genome suggest this conserved gene arrangement has either not been annotated properly or more likely has been deleted from these pared-down genomes entirely. The lack of an RNase P RNA raises several important questions. Is there a novel RNase P RNA in these obligate endosymbionts? Have they evolved a new process for the maturation of tRNA like *N. equitans*? Could they rely on the host mature tRNA for translation? Regardless of the answer, it will likely be a new evolutionary story in the bacterial kingdom.

## Conclusions

We have demonstrated that P Finder can rapidly and accurately identify all five structural types of RNase P RNA from large sequence data. Though previous *rnpB* annotation algorithms substantially advanced researchers’ capabilities of *rnpB* gene identification none could identify the more challenging structural types (T-type or C-type). P Finder, for the first time, can identify all five structural classes including T-type and C-type RNase P RNAs. With this capability P finder was used to scan completed genomes for RNase P RNA and identified only the second C-type RNase P RNA in *Sphaerobacter thermophilus.* Ten new T-type RNase P RNAs were also identified bring the total number of species with the minimal T-type RNase P RNA to 16.

The enigma of P continues with the inability of P Finder or other software programs to identify a *rnpB* gene in several bacterial obligate endosymbionts of sap sucking insects, Table 2. Due to the extremely small size of their genomes some researchers have suggested that they are more organelle than bacterial species. Similarly, *Nanoarchaeum equitans* also an obligate symbiont of the archaeon *Ignicoccus hospitalis. I. hospitalis* has a condensed genome (~500Kb) and is known *not* to encode an RNase P RNA*. N. equitans* is able to bypass the need for an RNase P RNA by transcribing its tRNA without a 5΄ leader sequence - as mature tRNA. It is possible that these obligate endosymbionts bypass the need for RNase P RNA by transcribing mature tRNAs lacking the leader sequence as well. Alternatively, it is also conceivable a new type or class of RNase P RNA is encoded within these genomes but is not yet detectable with currently computational methodologies.

## Availability

Project name: P Finder.

Project home page: https://github.com/JChristopherEllis/P-Finder

Operating system(s): Windows Vista/7/8/10.

Programming language: C#.

Other requirements: none.

License: BSD 3-clause “New” or “Revised” License.

Any restrictions to use by non-academics: license needed.

## Data Availability

All data was obtained from NCBI and is publicly available.

## Abbreviations

GUI: Graphical user interface
mRNA: Messenger RNA
ncRNA: Non-coding RNA
nt: Nucleotides
RNase P: Ribonuclease P
tmRNA: Transfer-messenger RNA
tRNA: Transfer RNA
